# Cyclodextrin Nanosponges Inclusion Compounds Associated with Gold Nanoparticles for Potential Application in the Photothermal Release of Melphalan and Cytoxan

**DOI:** 10.3390/ijms22126446

**Published:** 2021-06-16

**Authors:** Sebastián Salazar, Nicolás Yutronic, Marcelo J. Kogan, Paul Jara

**Affiliations:** 1 Departmento de Química, Facultad de Ciencias, Universidad de Chile, Las Palmeras 3425, Ñuñoa, Santiago 7800003, Chile; nyutroni@uchile.cl; 2 Departamento de Química, Farmacológica y Toxicológica, Universidad de Chile, Sergio Livingstone 1007, Santiago 8380492, Chile; 3 Advanced Center for Chronic Diseases (ACCDiS), Universidad de Chile, Santos Dumont 964, Independencia, Santiago 8380494, Chile

**Keywords:** β-cyclodextrin, cyclodextrin polymers, anti-tumor, drug delivery, photothermal therapy, plasmon surface resonance

## Abstract

This article describes the synthesis and characterization of β-cyclodextrin-based nano-sponges (NS) inclusion compounds (IC) with the anti-tumor drugs melphalan (MPH) and cytoxan (CYT), and the addition of gold nanoparticles (AuNPs) onto both systems, for the potential release of the drugs by means of laser irradiation. The NS-MPH and NS-CYT inclusion compounds were characterized using scanning electron microscopy (SEM), X-ray powder diffraction (XRPD), energy dispersive spectroscopy (EDS), thermogravimetric analysis (TGA), UV–Vis, and proton nuclear magnetic resonance (^1^H-NMR). Thus, the inclusion of MPH and CYT inside the cavities of NSs was confirmed. The association of AuNPs with the ICs was confirmed by SEM, EDS, TEM, and UV–Vis. Drug release studies using NSs synthesized with different molar ratios of β-cyclodextrin and diphenylcarbonate (1:4 and 1:8) demonstrated that the ability of NSs to entrap and release the drug molecules depends on the crosslinking between the cyclodextrin monomers. Finally, irradiation assays using a continuous laser of 532 nm showed that photothermal drug release of both MPH and CYT from the cavities of NSs via plasmonic heating of AuNPs is possible.

## 1. Introduction

In recent years, nanotechnology has received increasing attention and consideration due to its potential to improve the effectiveness of various drugs that are currently being used in cancer treatment. An inappropriate drug concentration at the tumor site and toxicity complications are the main causes of inadequate chemotherapeutic efficiency [1,2]. Localized delivery is imperative in order to develop efficient treatments for aggressive types of cancer, such as melanoma, ovarian cancer, neuroblastoma, and multiple myeloma, among others [3,4,5].

**Figure 1:**
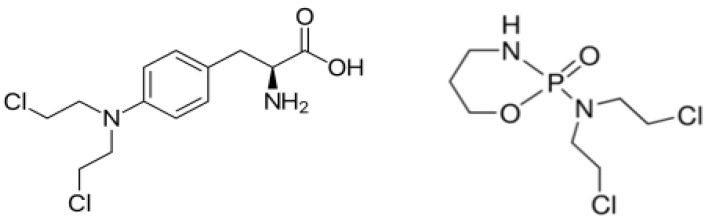
Chemical structure of MPH (left) and CYT (right).

Melphalan (MPH) and cytoxan (CYT) (Figure 1) are anti-cancer chemotherapy drugs classified as alkylating agents [5,6]. Alkylating agents work by damaging the DNA of cancer cells. Unfortunately, alkylating agents do not differentiate between cancerous cells and normal cells. Common side effects for patients taking MPH and CYT include low blood counts, nausea, vomiting, and hair loss, to name a few [7,8,9].

To overcome the limitations associated with anti-cancer agents and to minimize the adverse side effects, nano-materials and nano-porous systems (such as cyclodextrin-based nano-sponges (NS) have been synthesized and characterized, as they have great potential for applications in nano-therapies and drug delivery systems [10,11,12,13,14,15]. Cyclodextrins (CD) are water soluble oligosaccharides, most commonly consisting of six, seven, or eight glucopyranose units which are linked by α-1,4 glycosidic bonds. Among CDs, β-CD is interesting due to its cavity dimensions and its stability with crosslinking agents. As such, β-CD has been used to form nano-porous polymers [15]. The rising popularity of NSs as the material of choice over native CD is mainly due to their higher entrapment capacities, control over particle size and their ability to be regenerated and reutilized [16,17,18,19]. NSs can also improve the physicochemical properties of the guest molecules, such as solubility and stability, and absorb liquids into their nano-porous structure to convert liquid materials into a solid form [20].

NSs main advantages also include that they can be chemically modified and decorated with nanoparticles, thus improving the usefulness and properties of the polymer [21]. NSs have been proven to be safe, non-toxic, biodegradable, and of low cost [22,23,24]. As such, NSs have gained attention in targeted drug delivery research aimed at improving the efficacy of chemotherapies and reducing the adverse effects related to the drugs [16,17,18,20,25,26,27,28,29].

Gold nanoparticles (AuNP) also show promising characteristics in drug delivery systems, as they present high solubility, high stability in living systems and improved kinetics for drug distribution [30,31,32,33]. AuNPs can be conjugated to inclusion complexes (IC) of CDs if the guests have functional groups, such as thiols or amines [34,35]. NSs associated with AuNPs might promote the release of the included drugs by means of irradiation, due to the plasmon effect of AuNPs. A controlled drug delivery system using AuNPs and CD has been reported previously [36,37]; however, there are no such studies using systems with NSs as the matrix.

The objectives of this study were to synthesize and characterize NS inclusion compounds (IC) with the anti-tumor drugs MPH and CYT and to conjugate the ICs to AuNPs to study the migration of the guest molecules by laser irradiation. This article reports drug release studies using NSs inclusion compounds associated to AuNPs via plasmonic photothermia for the first time. These results could be of great interest as they contribute to the understanding of the potential utility of NSs associated to AuNPs for the controlled release of drug molecules via local photothermic effects.

## 2. Results and Discussion

### 2.1. Characterization of the ICs

#### 2.1.1. ^1^H-NMR Spectra of the ICs

**Figure 2:**
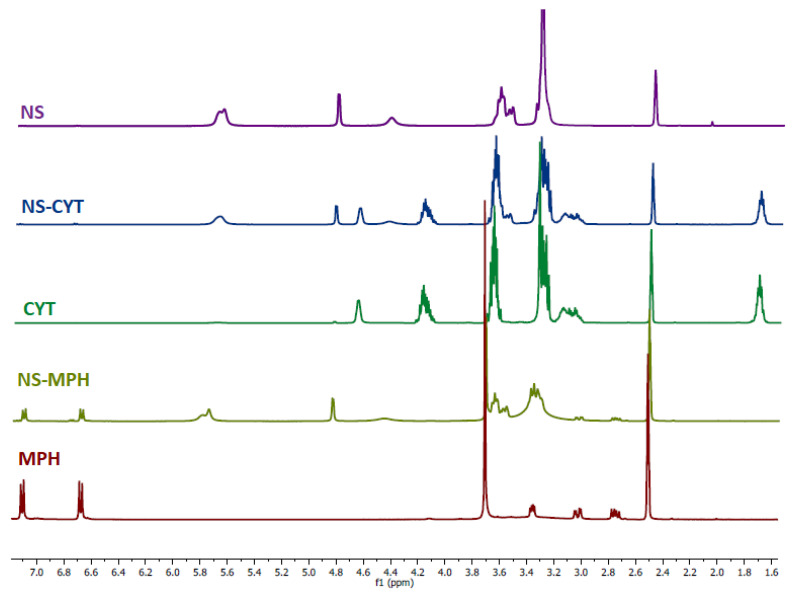
^1^H-NMR spectra of NSs, MPH, CYT, and the ICs.

To confirm the formation of the NS-MPH and NS-CYT complexes, ^1^H-NMR spectroscopy was used. The changes in the chemical shifts of the drugs provide evidence for the formation of the ICs. Figure 2 shows the acquired spectra of the NSs, the anti-tumor drugs and the ICs.

The proton signals of both guest molecules show high-field chemical shifts, possibly due to the screening effects caused by the spatial restriction and the change of environment due to the drugs being entrapped inside the cavities of the NSs. The protons within the hydrophobic cavities of the NSs (H_3_ and H_5_), as well as the hydroxyl groups (OH_2_ and OH_3_) displayed the most pronounced chemical shifts, which strongly suggests the complexation of the drugs. The protons on the outside cavities of the NSs (H_2_, H_4,_ and H_6_) also experienced chemical shifts. It is possible that the drugs were included not only inside the cavities of the CD monomers, but also on the multiple interstices of the NSs that were produced by polymerization. The differences in the chemical shifts of the drugs upon inclusion with NSs confirm the complexation of the drugs, as the occurrence of changes in the micro-environment between the free and entrapped drugs was expected, as reported in previous studies [11,21,38,39,40].

**Figure 3:**
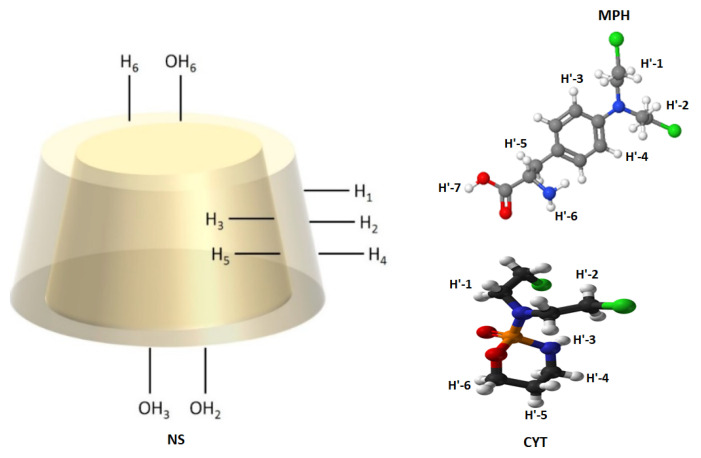
Proton assignments for the NSs, MPH, and CYT.

Proton assignments for the NSs and the anti-tumor drugs are shown in Figure 3. The chemical shifts for MPH and CYT after inclusion are shown in Table 1 and Table 2, respectively.

#### 2.1.2. TGA of the ICs

**Figure 4:**
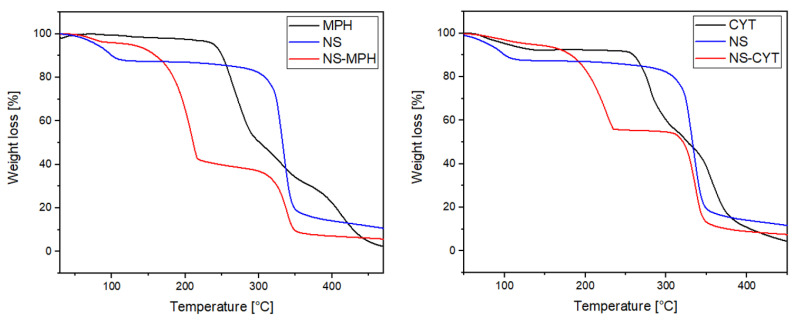
TGA of MPH, CYT, NS, and the ICs.

The TGA provides information about the thermal stability of the ICs in comparison to the free drugs. Figure 4 shows the TGA of MPH, CYT, NSs and the NSs loaded with the anti-tumor compounds.

MPH, CYT, NSs, and the ICs showed a weight loss at 100 °C due to the presence of water. NSs main degradation process occurs at 345 °C, which is an indicator of its good thermal stability. The main degradation step for MPH occurred at 250 °C, whereas for CYT, that weight loss was shown at 271 °C. The ICs’ thermogram shows two degradation steps. The first weight loss at 230 °C can be related to the degradation of the drugs. The second and main weight loss step was observed at 350 °C, which corresponds to degradation of NSs [38,41]. The thermograms show that the inclusion of MPH and CYT in the cavities of the NSs might increase the thermal stability of the drugs [21,29,41,42,43,44]. The decomposition temperatures for MPH, CYT and the ICs are summarized in Table 3.

#### 2.1.3. XRPD of the ICs

**Figure 5:**
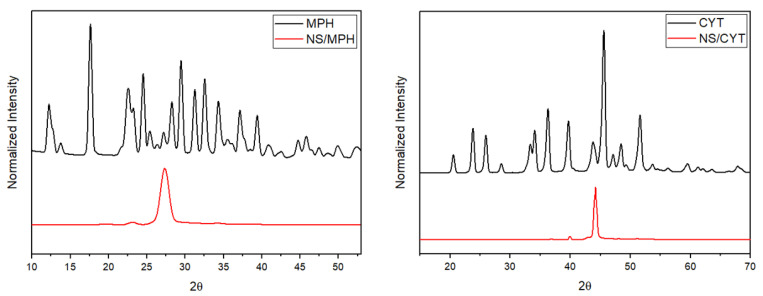
XRPD of MPH, CYT, and the ICs.

The XRPD analyses for MPH, CYT, and the ICs are shown in Figure 5. The XRPD results of the free drugs indicate strong diffraction peaks. The disappearance of these peaks after complexation of the drugs on the NSs indicates a reduction in the crystallinity of the drugs molecules. In the ICs diffractograms, a widened peak appeared due to a different crystal arrangement of the ICs. None of the characteristic peaks of the anti-tumor drugs are preserved in the NS-MPH and NS-CYT diffraction patterns. This confirms the encapsulation of the drugs inside the cavities of the NSs and the lack of free drugs in the ICs, as reported previously [21,38,45,46,47].

#### 2.1.4. SEM Analyses of the ICs

**Figure 6:**
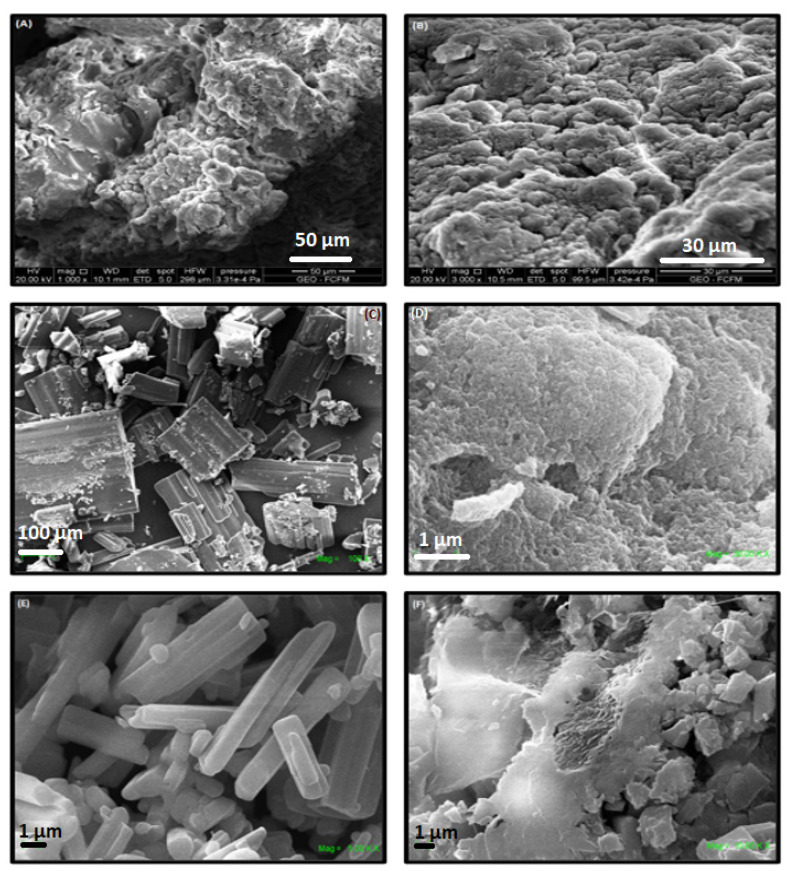
SEM micrographs of NSs (**A**,**B**), MPH (**C**), NS-MPH (**D**), CYT (**E**), and NS-CYT (**F**).

The formation of the ICs was confirmed using SEM and EDS analyses. Figure 6 shows SEM micrographs of free NSs, MPH, CYT, and the ICs. The images reveal the highly porous morphology of the NSs, whereas MPH and CYT show rectangular crystals and rods, respectively. After drug loading, changes in the surface of the samples were observed, as NSs show a homogeneous phase, with the absence of microcrystals of the drugs, confirming the inclusion of the guest molecules in the NSs cavities and excluding the possibility of co-precipitation of the individual phases [16,19,38,48].

#### 2.1.5. UV–Vis of MPH and CYT after Contact with NSs

**Figure 7:**
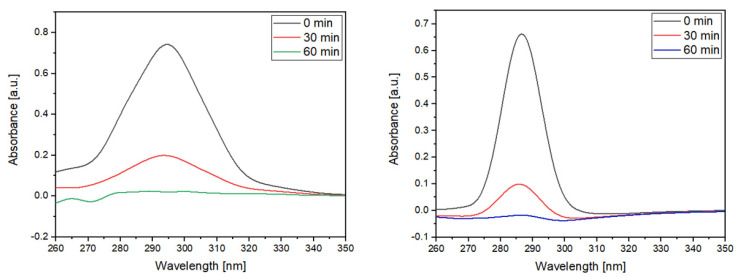
UV–Vis spectra of MPH (left) and CYT (right) after different contact times with NSs.

UV–Vis analyses were carried out in chloroform to determine the maximum absorbance of the drugs after different incubation times with the NSs. Figure 7 shows that the absorbance of both MPH and CYT decreased as the incubation time with the NSs increased, thus confirming the entrapment of the anti-tumor drugs inside the cavities of the polymer. The decrease in drug absorbance with increasing contact time of NSs indicates the formation of inclusion complexes, which are in a suspension, according to previous studies [21,28,49].

### 2.2. Characterization of ICs Associated with the AuNPs

#### 2.2.1. SEM and EDS Analyses of the ICs Associated with the AuNPs

**Figure 8:**
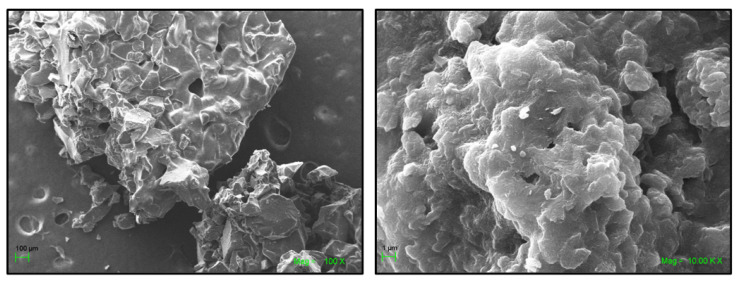
SEM micrographs of NS-MPH associated with the AuNPs.

**Figure 9:**
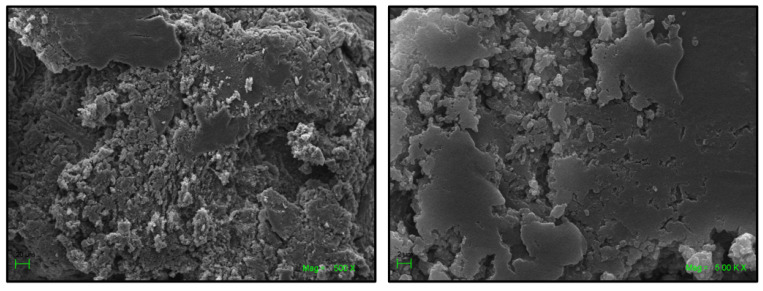
SEM micrographs of NS-CYT associated with the AuNPs.

Figure 8 and Figure 9 show SEM micrographs of the AuNPs attached to the ICs’ surface. It is evident that the ICs retained their porous morphology after the deposition of the gold nanoparticles.

**Figure 10:**
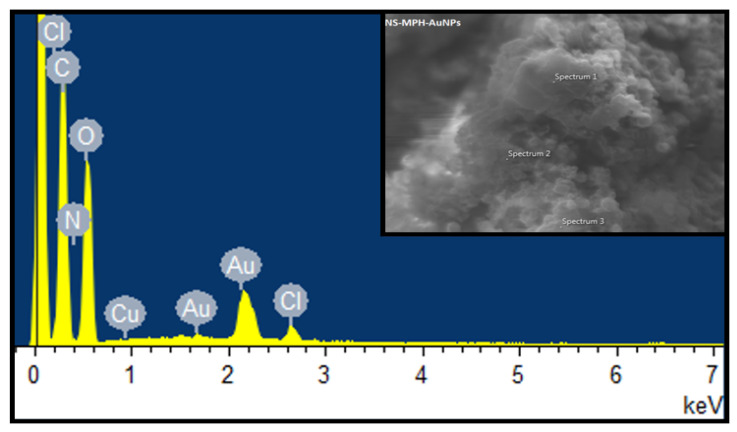
EDS analysis of NS-MPH associated with the AuNPs.

**Figure 11:**
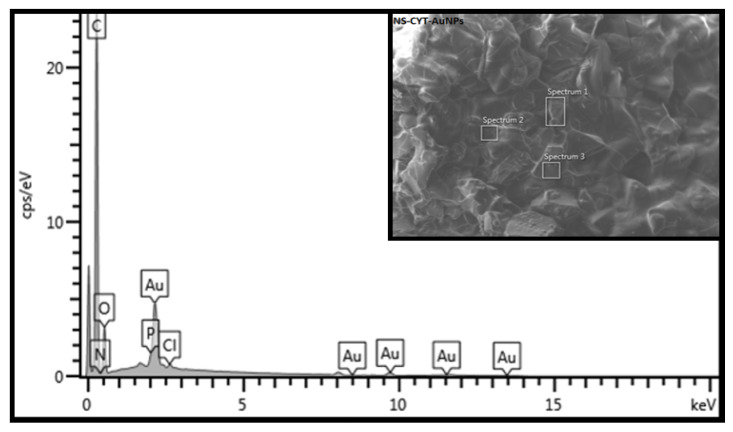
EDS analysis of NS-CYT associated with the AuNPs.

The EDS analysis results are shown in Figure 10 and Figure 11 for NS-MPH-AuNP and NS-CYT-AuNP, respectively. These analyses provide information about the elemental composition of the ICs. The graphs show the weight percentages and presence of C, O, N and Cl in the NS-MPH complex. N and Cl correspond to the amine and chloroethylamine functional groups of MPH. The EDS result of NS-CYT shows the detection of C, O, N, P, and Cl due to the amine, phosphate and chloroethyl functional groups of CYT. The EDS spectra in Figure 9 and Figure 10 also show the detection of Au, which confirms the association of the ICs to the AuNPs.

#### 2.2.2. TEM Analyses of the ICs Associated with the AuNPs

**Figure 12:**
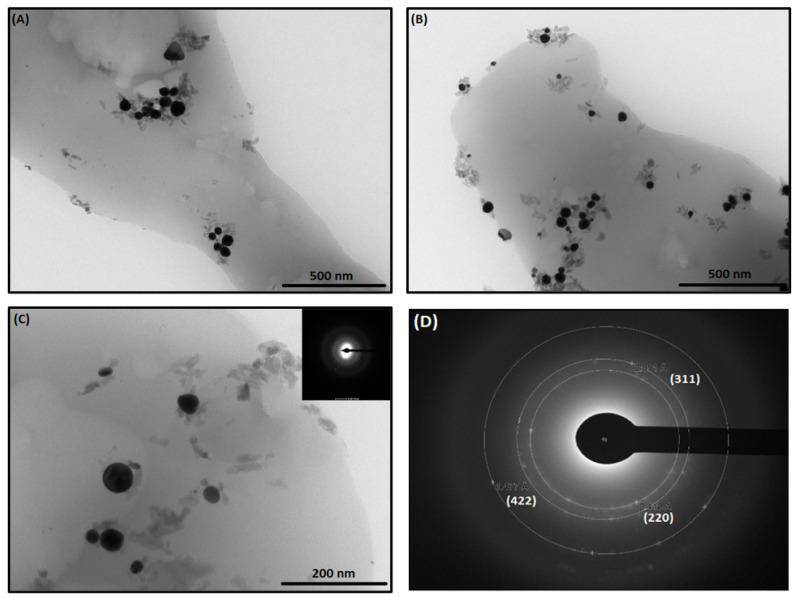
TEM micrographs of NS-MPH associated with AuNPs (**A**), NS-CYT associated with AuNPs (**B**), NS-CYT associated with AuNPs selected area for SAED (**C**), and SAED pattern of AuNPs (**D**).

TEM micrographs of the ICs conjugated to the AuNPs are shown in Figure 11. The AuNPs are distributed on the ICs, both isolated and aggregated, contained in the organic material. The micrographs show three components in the ICs: the spherical AuNPs, the organic phase corresponding to the ICs, and a nano bar-shaped component associated to the AuNPs. The nano bars are of organic nature, which can probably be associated to the crystallization of the anti-tumor drugs, due to a partial disintegration of the ICs during preparation of the TEM samples with ethanol. Selected area electron diffraction (SAED (Figure 12D)) confirms a single metallic crystalline phase which can be indexed to the (220), (311), and (422) crystallographic planes of AuNPs [50].

#### 2.2.3. UV–Vis Spectra of the ICs Associated with the AuNPs

**Figure 13:**
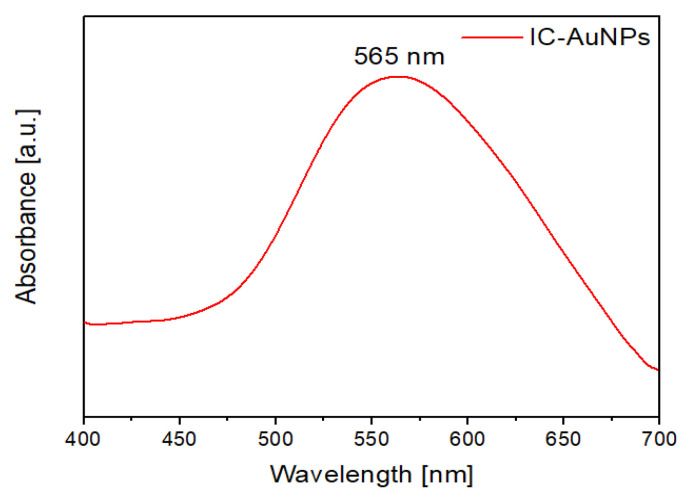
UV–Vis spectra of the AuNPs deposited on the ICs’ surface.

UV–Vis and diffuse reflectance are useful techniques to confirm the deposition of nanoparticles onto the surface of organic substrates. Figure 13 shows the absorption spectra of the ICs conjugated to the AuNPs. The characteristic plasmon band of spherical gold nanoparticles was observed at 565 nm. The bathochromic shift could be due to the aggregation of some of the AuNPs onto microcrystals, which is consistent with the broadening of the absorption band and with the TEM images shown in Section 2.2.2. The presence of the plasmon band in the ICs conjugated to the AuNPs provides evidence of the nanoparticles’ stability, as indicated by previous studies [36,37].

#### 2.2.4. DLS and Z-Potential of the ICs Associated with the AuNPs

Table 4 shows the hydrodynamic diameter and Z-Potential of the AuNPs and the ICs conjugated to the AuNPs.

The AuNPs showed a Z-Potential of −46 mV. The negative surface charge can be attributed to the stabilization by the citrate ions. Colloidal AuNPs show a hydrodynamic diameter of 19 nm, which is different than the diameter of 12 nm shown by TEM, as the latter technique indicates the diameter of the metallic phase. DLS also provides information of the hydrodynamic diameter of the ternary systems, with depicted values of 633 for AuNPs-NS-MPH and 618 for AuNPs-NS-CYT. Upon association, the Z-Potential of the AuNPs decreased due to the stabilization of the AuNPs by the neutral ICs. Nano-suspensions with Z-Potential values over ±30 mV are considered stable according to the literature [20,29,51], thus confirming that the supramolecular systems will not undergo aggregation over time. Polydispersity index (PDI) values are used to describe the size distribution of nanoparticles. PDI values over 0.7 indicate that the samples have broad particle size distribution [13]. Thus, the obtained PDI values for AuNPs and the AuNPs associated to the ICs show that the nano formulations are stable and homogeneous in nature.

### 2.3. Guest Photothermal Release by Laser Irradiation

#### 2.3.1. Drug Loading and Encapsulation Efficiencies

MPH and CYT were loaded in NSs with different β-CD and DPC molar ratios (1:4 and 1:8). The complexes’ stoichiometry was determined using the ^1^H-NMR spectra of the ICs and the free drugs, with the integration of H1 of the NSs as a reference. This allowed for the determination of host numbers per drug [52]. The observed NS/drug ratios were 1:7 for the drugs loaded in NSs (1:4) and 1:4 for the drugs loaded in NSs (1:8). Drug loading and encapsulation efficiencies were calculated using Equations (1) and (2), respectively. Among the NSs, the drug loading and encapsulation efficiency was higher in NSs (1:4) than in NSs (1:8), as shown in Table 5. This indicates that the degree of crosslinking in the polymer affected the complexation capacity of the NSs.

Crosslinking on NSs with different molar ratios can be estimated using ^1^H-NMR. The technique allows to compare the integration of the hydroxyl groups of native β-CD with the integration of the hydroxyl group of NSs. Integration deltas of OH groups increase as the concentration of crosslinker increases, as they react with DPC to form links between the CD monomers. These results are shown in Table 6, thus confirming the higher degree of crosslinking on NSs (1:8).

#### 2.3.2. Laser Irradiation Assays

**Figure 14:**
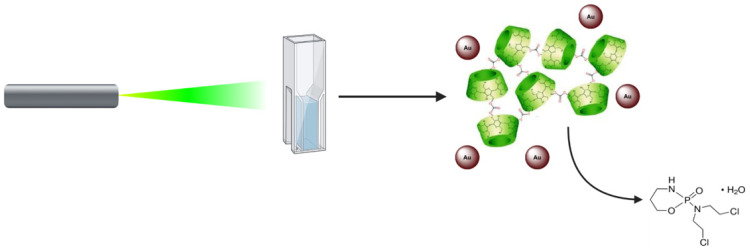
Schematic representation of guest molecule migration by means of plasmonic hyperthermia.

To study drug migration phenomena by laser irradiation, the ICs associated with the AuNPs were added to a two-phase system consisting of an aqueous phase and an organic phase. Generation of local heat might promote the release of MPH and CYT molecules, as shown in Figure 14.

The assays were performed by irradiation with a laser at intervals of 15 min until a maximum time of 90 min was reached. Absorbance of the guest molecules was measured using UV–Vis in chloroform. The concentration of the drugs was determined using the Lambert–Beer equation. Molar attenuation of both drugs was determined using a set of drug solutions using UV–Vis spectroscopy. The molar attenuation was 9.31 mM^−1^ cm^−1^ for MPH and 7.27 mM^−1^ cm^−1^ for CYT.

Drug release assays were carried out using NSs synthesized with different molar ratios of β-CD and DPC (1:4 and 1:8) to determine which system provides higher loading efficiencies and drug release percentages.

**Figure 15:**
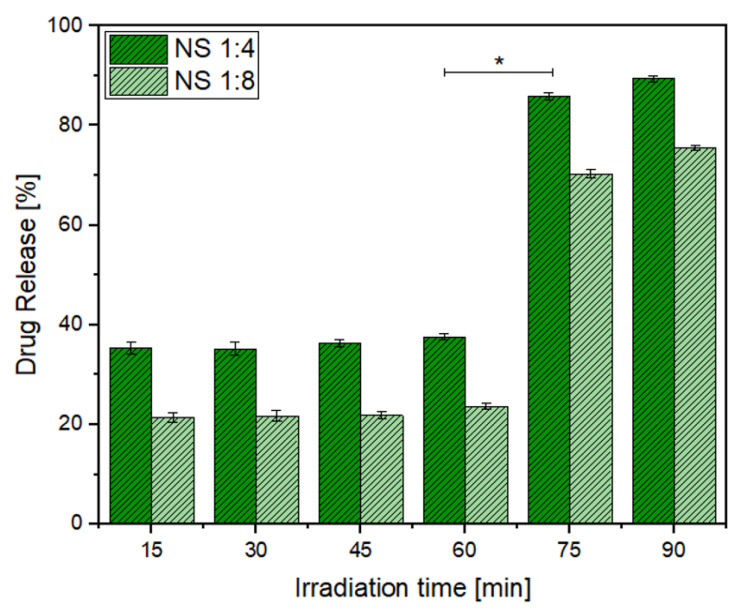
Drug release percentages for MPH. Statistical analysis from ANOVA–Tukey (* *p* < 0.05 versus NS 1:8).

**Figure 16:**
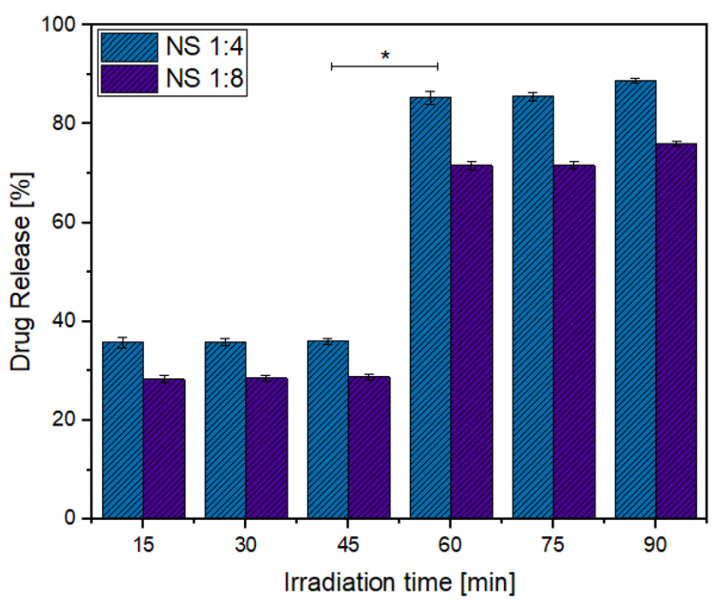
Drug release percentages for CYT. Statistical analysis from ANOVA-Tukey (* *p* < 0.05 versus NS 1:8).

Figure 15 and Figure 16 show the percentages of drug released to the organic phase at different irradiation times for MPH and CYT, respectively.

Migration of the drugs to the organic phase occurred gradually, as the release of both drugs was observed from the first interval at 15 min. The highest drug release percentage was observed at 60 min for CYT and at 75 min for MPH, suggesting that those are the optimum times for irradiation. After the optimum irradiation time, the concentration of released drug increased only slightly.

Drug release profiles for both MPH and CYT take place in two steps. The first step could be related to the guest molecules that are entrapped at the surface of the NSs, making them the first to be released. The second step corresponds to the guest molecules that are included within the NS matrix and, thus, might have higher affinity to the NSs than the surface molecules [21,28].

NSs (1:4) showed higher drug release percentages at all irradiation times in comparison to NSs (1:8). The differences in the release patterns show that the migration of the guest molecules and the ability of NSs to entrap the drug molecules depend on the crosslinking between the cyclodextrin monomers. A higher concentration of cross-linker results in hindering of the interaction of MPH and CYT with the cavities of the NSs, thus reducing their effectiveness as a drug carrier [53,54].

**Figure 17:**
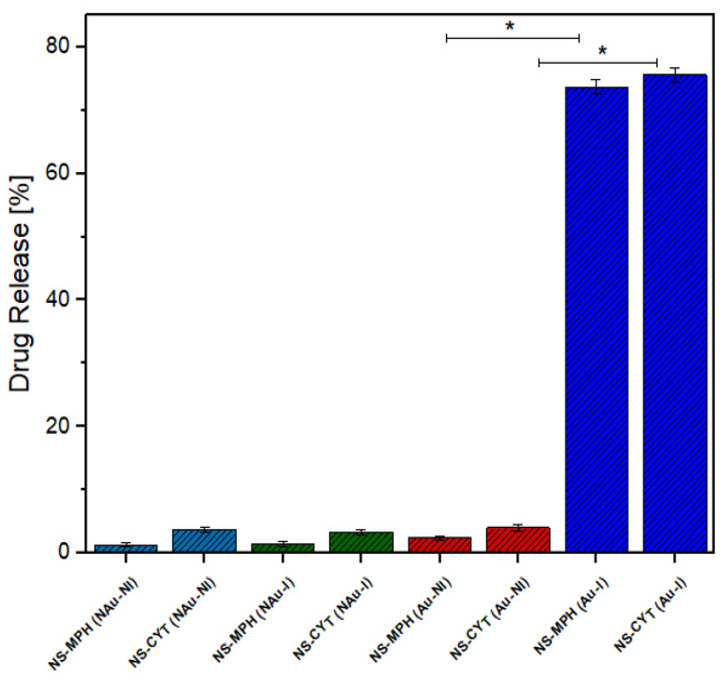
Drug release (%) for MPH and CYT after 90 min of irradiation. NAu-Ni = No AuNPs and no irradiation. NAu-I = No AuNPs and irradiation. Au-I = AuNPs and irradiation. Statistical data analysis from ANOVA–Tukey (* *p* < 0.05 versus Au-NI).

The IC-AuNP system was compared with an IC-AuNP system without irradiation and with a system consisting of ICs without nanoparticles in order to determine whether the anti-tumor drugs migrate to the organic phase of the system by means of diffusion. Figure 17 shows the drug release percentages for MPH and CYT after an irradiation time of 90 min, for the above-mentioned ICs.

Figure 16 shows that the amount of drug released from the ICs without AuNPs was drastically reduced in comparison to the irradiated NSs that were conjugated to the AuNPs. This shows that the local hyperthermia produced by AuNPs causes the release of the drugs to be faster and more efficient. No burst effect was observed on the release profiles, indicating that the NSs could be a potential solution for dose-related side effects.

## 3. Materials and Methods

### 3.1. Materials

All chemical reactants used in this study are commercially available and were used as received: β-cyclodextrin (Sigma-Aldrich, Saint Louis, MO, USA), melphalan (Sigma-Aldrich), cytoxan (Sigma-Aldrich), diphenylcarbonate (Sigma-Aldrich), chloroauric acid (Sigma-Aldrich), sodium citrate (Sigma-Aldrich) and nano-pure water (Merck, Darmstadt, Germany). The glassware used for the synthesis and sorption studies was washed with aqua regia (3 HCl:1 HNO_3_), and then rinsed repeatedly with Milli-Q water.

### 3.2. Synthesis of AuNPs

AuNPs were synthesized according to the Turkevich method [55,56]. In a 200-milliliter flask equipped with a condenser, 100 mL of a 1 mM HAuCl_4_ aqueous solution was brought to boil under stirring conditions. Then, 10 mL of a 38.8 mM solution of sodium citrate was added to the HAuCl_4_ solution as quickly as possible. The solution was heated for 30 min and then allowed to cool. Then, the solution was filtered using a 0.45-micrometer cellulose acetate membrane filter, thus obtaining AuNPs of 12 nm in diameter.

### 3.3. Synthesis of the NSs

The synthesis of the NSs was adopted from a published procedure [10] with slight modifications. The NSs were prepared using 1.5 g of β-CD/0.856 g of diphenylcarbonate (DPC) (molar ratio of 1:4) and 1.5 g of β-CD/1.72 g of DPC (molar ratio of 1:8). Homogenized anhydrous β-CD and DPC were placed in a conical flask. The reactants were heated to 100 °C under magnetic stirring and left to react for 6 h. During the reaction, phenol crystals appeared in the neck of the flask. The reaction mixture was left to cool, and the obtained white powder was broken up roughly with an agate mortar. The solid was repeatedly washed with distilled water to remove unreacted CD and with acetone to remove unreacted DPC and phenol, which was a byproduct of the reaction. Afterwards, the solid was further washed by Soxhlet extraction with ethanol for 48 h. Finally, the solid was left to dry at 100 °C in an oven for 48 h and stored at 25 °C for further use. Complete characterization of NSs was carried out by FT-IR, SEM, TEM, TGA, ^1^H-NMR, DLS, XRPD, and BET analysis using previously reported methods [38].

### 3.4. Preparation of NS-MPH and NS-CYT ICs

MPH and CYT were loaded in NSs using previously reported methods [57]. Briefly, 1 g of NS was dispersed in 50 mL of double-distilled water using a magnetic stirrer, and then, 20 mL each of MPH and CYT was added. The mixture was sonicated for 10 min and then kept for 24 h under stirring. The suspensions were centrifuged at 3000 rpm for 15 min to separate uncomplexed drugs from the colloidal supernatant. The obtained supernatant was lyophilized at −81 °C and 0.001 mbar. The dried powder was stored in a desiccator for further use.

### 3.5. Association of AuNPs onto the ICs

Briefly, 0.3 g of the ICs was immersed in 600 µL of AuNPs. The suspension was allowed to settle for 20 min and then centrifuged at 20,000 rpm for 30 min. Finally, the AuNPs associated with the ICs were dried under vacuum for further use.

### 3.6. Proton Nuclear Magnetic Resonance (^1^H-NMR) Spectroscopy

^1^H-NMR spectra of the NSs, MPH, CYT and the ICs were obtained using a Bruker Advance 400 MHz spectrometer. Stock solutions of the NSs, pesticides and complexes were prepared in deuterated dimethyl sulfoxide (DMSO) with tetramethyl silane (TMS) as an internal reference.

### 3.7. X-ray Powder Diffraction (XRPD)

XRPD patterns of the NSs and the ICs were recorded using a high-power powder Siemens/Bruker D5000 diffractometer equipped with a Cu anode and a Ni target filter. The diffractometer had a current of 40 kV/40 mA and a scan speed of 0.05°/s. The samples were analyzed over a 2ϴ angle range of 2–30°.

### 3.8. Thermogravimetric Analysis (TGA)

TGA of the NSs, MPH, CYT, and the ICs was performed on a TGA-4000 Pyris 6 thermogravimetric analyzer over a temperature range of 25 to 400 °C. Approximately 10 mg of the sample was placed on a pan, which was then subjected to the above-mentioned conditions under nitrogen atmosphere.

### 3.9. Scanning Electron Microscopy (SEM)

The surface morphology of the NSs, MPH, CYT, the ICs and the AuNPs conjugated to the ICs was determined using a LEO VP1400 analytical scanning electron microscope equipped with an EDS. The samples were prepared by application to carbon films coated on aluminum stubs.

### 3.10. Ultraviolet and Visible Absorption (UV–Vis) Spectroscopy

UV–Vis spectra of the antitumor drugs and the AuNPs conjugated to the ICs were recorded using a Jasco V-760 UV–Vis spectrometer. Measurements were carried out over a range of 300 to 700 nm, using deionized water as a reference and concentrations of 0.01 mol/L for each drug.

### 3.11. Transmission Electron Microscopy (TEM)

TEM analyses were performed using a Zeiss model EM-109 microscope, operating at 50 kV. All the samples were prepared by depositing 20 µL of the solution on a copper grid with a film of Formvar.

### 3.12. Determination of Drug Content on NSs

The encapsulation efficiency and drug loading capacity were calculated using the following equations [20]:

(1) $$\mathrm{Drug}\;\mathrm{loading}=\;\frac{\mathrm{weight}\;\mathrm{of}\;\mathrm{drug}\;\mathrm{in}\;\mathrm{NSs}}{\mathrm{weight}\;\mathrm{of}\;\mathrm{NSs}}\;\times \;100\%$$

(2) $$\mathrm{Encapsulation}\;\mathrm{efficiency}=\;\frac{\left[\mathrm{Drug}\right]\;\mathrm{in}\;\mathrm{NSs}}{\left[\mathrm{Drug}\right]\;\mathrm{initially}}\;\times \;100\%$$

### 3.13. DLS and Z-Potential

Hydrodynamic radius and Z-Potential were calculated using a DLS Zetasizer NanoS series, Malvern. All samples were diluted with double-distilled water before each measurement. Z-Potential measurements were made at a constant temperature of 25 °C using disposable zeta cells. A total of 12 measurements were carried out, and their average is reported in the results.

### 3.14. Laser Irradiation Assays

Irradiation assays were carried out in accordance with previous studies [36,58,59]. A continuous laser at 532 nm with a beam diameter of 1 mm and 80 mW of light power was used. Due to the low aqueous solubility of both drugs, a system consisting of two phases was formed, using quartz cuvettes with 0.3 mL of a chloroform solution and 0.3 mL of water. Then, the ICs conjugated to the AuNPs were added. The AuNPs on the surface of the ICs were exposed to laser irradiation at different times (intervals of 15 min until a maximum time of 90 min was reached). The absorbance of both MPH and CYT was measured on the organic phase using UV–Vis. ICs without AuNPs were used for laser irradiation analyses in order to determine whether AuNPs are responsible for the drugs’ release. The maximum absorbances for the guest molecules were converted to drug release percentages and then compared with the total amount of drug that was aggregated in the system. All independent experiments were performed in triplicate. Percentages of released drug on the organic phase were calculated using the following equation:

(3) $$\mathrm{Drug}\;\mathrm{Release}\;\left(\%\right)=\;\frac{\left[\mathrm{Drug}\right]\;\mathrm{after}\;\mathrm{irradiation}}{\left[\mathrm{Drug}\right]\;\mathrm{added}\;\mathrm{initially}\;}\;\times \;100\%$$

### 3.15. Temperature Control of the Samples after Irradiation

The ICs conjugated to the AuNPs (20 mg) were added to 0.5 mL of a chloroform solution. Afterwards, the mixture was irradiated with a continuous laser beam at 532 nm. The temperature of the system after irradiation was measured at different times (intervals of 15 min until a maximum time of 120 min was reached) using a digital thermometer. The highest temperature reached was 31 °C at 120 min, whereas the minimum temperature was 26 °C at 0 min (no irradiation). This confirmed that the temperature of the solution does not significantly increase after irradiation, which is in agreement with previous studies [59].

### 3.16. Data Analysis

The experiments were performed in triplicate, and the results are reported as mean ± SD. Statistical measurements were performed using GraphPad Prism 5 Software Inc. (San Diego, CA, USA). A one-way ANOVA with Tukey’s test was used, and results were considered to be significant if *p* < 0.05.

## 4. Conclusions

In this work, we successfully promoted the release of MPH and CYT from the cavities of NSs via plasmonic heating of the AuNPs that were conjugated to the ICs. The β-CD-based NSs were able to efficiently form inclusion complexes with MPH and CYT, as proven using SEM, TGA, ^1^H-NMR, XRPD and UV–Vis characterization. The SEM, EDS, TEM, DLS, Z-Potential, and UV–Vis analyses showed that the ICs were a great substrate for stabilizing the AuNPs, thus giving the polymer additional and useful properties, as decorated NSs clearly outperform native NSs in terms of drug release efficiency. It is important to note that the guest molecule release not only depends on the conjugation of the ICs to the AuNPs, but also on the degree of crosslinking of the polymer, as a higher concentration of cross-linker could result in reduced availability of the inclusion sites. NS materials conjugated to nanoparticles could eventually be considered an improved technology for drug delivery, as they are cheap, efficient, and non-toxic materials.

## Figures and Tables

**Table:** 

System	H1	H2	H3	H4	H5	H6	OH2	OH3	OH6
NS	4.827	3.300	3.627	3.361	3.579	3.655	5.705	5.673	4.440
NS-MPH	4.825	3.298	3.618	3.358	3.573	3.652	5.718	5.680	4.445
Δδ	0.002	0.002	0.009	0.003	0.006	0.003	−0.013	−0.007	−0.005

**Table:** 

System	H’1	H’2	H’3	H’4	H’5	H’6	H’7
MPH	3.738	3.447	7.135	6.798	2.835	3.117	11.035
NS-MPH	3.735	3.444	7.130	6.793	2.830	3.115	11.029
Δδ	0.003	0.003	0.005	0.005	0.005	0.002	0.006

**Table:** 

System	H1	H2	H3	H4	H5	H6	OH2	OH3	OH6
NS	4.827	3.300	3.627	3.361	3.579	3.655	5.705	5.673	4.440
NS-CYT	4.822	3.297	3.621	3.359	3.572	3.652	5.712	5.678	4.443
Δδ	0.005	0.003	0.006	0.002	0.007	0.003	−0.007	−0.005	−0.003

**Table:** 

System	H’1	H’2	H’3	H’4	H’5	H’6
CYT	3.380	3.735	4.228	1.728	3.465	3.319
NS-CYT	3.378	3.733	4.225	1.725	3.460	3.316
Δδ	0.002	0.002	0.003	0.003	0.005	0.003

**Table 3:** Decomposition temperatures and weight loss for MPH, CYT, and the ICs.

System	Decomposition Temperature (°C)	Weight Loss (%)
NS	345.7	65.1
MPH	249.5	25.1
NS-MPH	233.9	39.7
NS-MPH	348.1	48.3
CYT	271.5	27.1
NS-CYT	235.1	41.1
NS-CYT	350.7	49.9

**Table 4:** DLS and Z-Potential of AuNPs and ICs associated with the AuNPs.

System	DLS (nm)	Z-Potential (mV)	PDI
AuNPs	19 ± 2	−46 ± 0.4	0.21
AuNP-NS-MPH	633 ± 43	−31 ± 0.5	0.47
AuNP-NS-CYT	618 ± 38	−35 ± 0.5	0.45

**Table 5:** Drug loading and encapsulation efficiencies for NS-MPH and NS-CYT.

System	Drug Loading (%)	Encapsulation Efficiency (%)
NS (1:4)–MPH	83.1 ± 2.25	90.3 ± 0.25
NS (1:4)–CYT	85.2 ± 2.31	93.7 ± 0.22
NS (1:8)–MPH	63.1 ± 2.13	75.3 ± 0.15
NS (1:8)–CYT	67.2 ± 2.08	77.1 ± 0.35

**Table 6:** Chemical shifts and integrations of NSs (1:4), NSs (1:8) and free β-CD.

Protons	β-CD	NSs 1:4	NSs 1:8	∫ (β-CD)	∫ (NSs 1:4)	∫ (NSs 1:8)	Δ∫ (NSs 1:4)	Δ∫ (NSs 1:8)
OH2	5.721	5.705	5.701	7.03	6.33	5.67	0.7	1.36
OH3	5.670	5.673	5.670	7.05	6.22	5.40	0.83	1.65
OH6	4.453	4.440	4.437	7.15	6.18	5.33	0.97	1.82

## Abbreviations

DMSO	Dimethyl sulfoxide
TMS	Tetramethylsilane
NSs	Nanosponges
DPC	Diphenylcarbonate
β-CD	Beta cyclodextrin
IC	Inclusion compound
MPH	Melphalan
CYT	Cytoxan
AuNPs	Gold nanoparticles
^1^H-NMR	Proton nuclear magnetic resonance
TGA	Thermogravimetric analysis
DLS	Dynamic light scattering
XRPD	X-ray powder diffraction
SEM	Scanning electron microscopy
TEM	Transmission electron microscopy
EDS	Energy dispersive spectroscopy
SAED	Selected area electron diffraction
PDI	Polydispersity index
